# Experimental Selection for *Drosophila* Survival in Extremely High O_2_ Environments

**DOI:** 10.1371/journal.pone.0011701

**Published:** 2010-07-23

**Authors:** Huiwen W. Zhao, Dan Zhou, Victor Nizet, Gabriel G. Haddad

**Affiliations:** 1 Divisions of Respiratory Medicine, Infectious Disease and Pharmacology and Drug Discovery, Department of Pediatrics, University of California San Diego, La Jolla, California, United States of America; 2 Skaggs School of Pharmacy and Pharmaceutical Sciences, University of California San Diego, La Jolla, California, United States of America; 3 Rady Children's Hospital, San Diego, California, United States of America; Centre for Genomic Regulation (CRG), Universitat Pompeu Fabra, Spain

## Abstract

Although oxidative stress is deleterious to mammals, the mechanisms underlying oxidant susceptibility or tolerance remain to be elucidated. In this study, through a long-term laboratory selection over many generations, we generated a *Drosophila melanogaster* strain that can live and reproduce in very high O_2_ environments (90% O_2_), a lethal condition to naïve flies. We demonstrated that tolerance to hyperoxia was heritable in these flies and that these hyperoxia-selected flies exhibited phenotypic differences from naïve flies, such as a larger body size and increased weight by 20%. Gene expression profiling revealed that 227 genes were significantly altered in expression and two third of these genes were down-regulated. Using a mutant screen strategy, we studied the role of some altered genes (up- or down-regulated in the microarrays) by testing the survival of available corresponding P-element or UAS construct lines under hyperoxic conditions. We report that down-regulation of several candidate genes including *Tropomyosin 1*, *Glycerol 3 phosphate dehydrogenase*, *CG33129*, and *UGP* as well as up-regulation of *Diptericin* and *Attacin* conferred tolerance to severe hyperoxia. In conclusion, we identified several genes that were not only altered in hyperoxia-selected flies but we also prove that these play an important role in hyperoxia survival. Thus our study provides a molecular basis for understanding the mechanisms of hyperoxia tolerance.

## Introduction

Mammalian respiratory, cardiovascular and neurologic systems tightly regulate oxygen (O_2_) levels. Although O_2_ is essential for aerobic life, levels that are either too low or too high can induce morbidity and mortality. For this reason, O_2_ has often been considered as both an elixir and a poison.

Except possibly in skin (e.g., wound healing), high O_2_ induces oxidant injury in almost every organ, especially in the lung, retina, heart and brain [1], [2], [3]. Prolonged exposure to high oxygen generates excessive reactive oxygen species, induces cell death and oxidative stress responses, affects immune response and DNA integrity and modulates cell growth [4], [5], [6], [7], [8]. Disorders including neurodegenerative and chronic inflammatory diseases, as well as damage from ischemia and consequent reperfusion to the heart, lung, retina, brain, and other organs result, by and large, from oxidant injury. Mammalian aging can also be attributed, at least in part, to oxidant injury. Numerous studies have defined the phenotype of hyperoxia-induced injury and explored the underlying mechanisms, especially in lung and retina [5], [9], [10], [11], [12], [13], [14]. For example, proinflammatory cytokines, such as IL-8, IL-1, IL-6, IL-13 and many signaling systems, including the JNK, ERK, NF-kappa B, and TGF-beta pathways, appear to play a role in the genesis of hyperoxic injury. In addition, the newborn mammal has been shown to be more resistant to hyperoxia challenge than the adult, possibly reflecting differing O_2_ defense mechanisms. However, conclusions drawn from many of these studies are at best correlative.

The importance of oxidative stress is not limited to mammalian tissues. *Drosophila melanogaster* has similar O_2_ response pathways to those of mammals and research on flies has enhanced our understanding of oxidant stress [15], [16], [17], [18]. In our laboratory, we have used this model organism to investigate the tolerance and susceptibility to O_2_ [17], [19], [20]. To study the mechanisms underlying hyperoxia tolerance, we have generated a *Drosophila melanogaster* strain that is extraordinarily resistant to high O_2_ levels through laboratory selection over many generations. These flies are now living and reproducing at ∼90% O_2_ without any overt signs of injury. In the current study, we used this novel hyperoxia-tolerant strain as a model to identify differentially expressed genes that are altered by hyperoxia selection and to investigate the role of single genes that may functionally contribute to hyperoxia tolerance.

## Results

### Hyperoxia selection changes the fly phenotype

To investigate hyperoxia sensitivity and tolerance, we used a similar paradigm to the one we previously described [21] to generate a *Drosophila* strain that tolerates extremely high oxygen conditions after a long term experimental selection. In brief, we first pooled 27 wild-type isogenic lines to construct a parental population of *Drosophila melanogaster* and then collected and cultured F1 embryos of this pooled population in one of six separate chambers under different levels of O_2_: in normoxia (21% O_2_) as a control (three chambers) and in hyperoxia (three chambers) for the experimental selection. The levels of hyperoxia-tolerance in the 27 parental lines were tested individually under 60%, 70%, 80% or 90% O_2_. At levels under 60% O_2_ we found that embryos from most parental lines reached the adult stage, but with a reduced population size. At 80% O_2_ and above, the growth of all parental lines was eliminated. Hyperoxia selection was therefore initiated at 60% O_2_ with a gradual increase in O_2_ concentration, every 3–5 generations, to maintain the selection pressure. By the 13^th^ generation, we obtained flies that were able to live perpetually in 90% O_2_. Although severe hyperoxic environments were known to reduce lifespan in flies, the embryos from S_O2_A flies were able to complete development to adult stage and reproduced indefinitely in 90% O_2_. In contrast, embryos from the control population stopped development in 90% O_2_ at the 1^st^ instar larval stage, failed to molt, became necrotic, and died.

**Figure 1:**
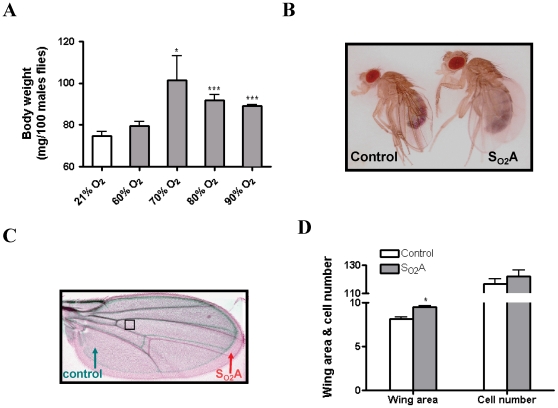
Phenotypic changes following long-term hyperoxia selection. A) Three sets of 100 hyperoxia–selected (S_O2_A) male flies, which were perpetually living in 90% O_2_, were collected from each chamber and weight was averaged. S_O2_A flies (grey bar) have an increased body weight as compared to control flies (white bar) when O_2_ concentration was 70% (p<0.05) or above (p<0.001). B) A representative image from control flies and S_O2_A flies is shown in Fig. 1B. C & D) Wings from 12 male flies in each group were collected and imaged by an Axiovert 200 M microscope. Wing area and cell number were determined using Image J. A representative picture of wings from a control (green) and a S_O2_A (red) fly was shown in Fig. 1C, cell number was measured in the area of wings shown in square. S_O2_A flies had a larger wing area than control flies (p<0.05) and the cell number per area was not different between two groups. * indicates p<0.05; ** indicates p<0.01; *** indicates p<0.001.

Hyperoxia-selected adult flies differed from control flies in morphology and size. Body weight of S_O2_A flies, which tolerated 90% O_2_, was at least 20% greater than that of control flies when the O_2_ concentration was 70% (p<0.05) or higher (p<0.001) (Fig. 1A). S_O2_A flies also had a larger body size and larger wing area than flies propagated in normoxia (Fig. 1B & C). However, cell number per area was not different between two groups (Fig. 1D) indicating that S_O2_A flies have an increased total cell number when compared to control flies.

**Figure 2:**
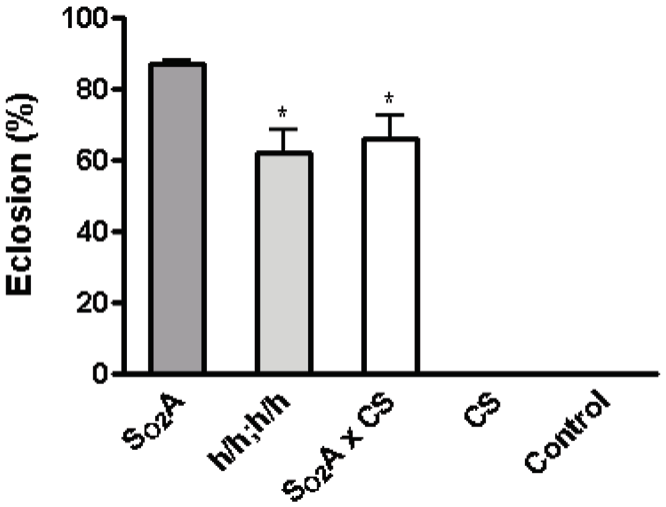
Hyperoxia tolerance is a heritable trait. Embryos from S_O2_A, *h/h; h/h* (progeny of a cross among *h/SM5; h/TM3 sb* flies), *S_O2_A x CS* (progeny of a cross between S_O2_A flies and *CS* flies), *CS*, and control flies were collected and cultured in 90% O_2_. Their percent eclosion was compared. About 65% of the pupae from *h/h; h/h* and *S_O2_A x CS* lines eclosed in 90% O_2_ as compared to 88% eclosion for S_O2_A flies (p<0.05) and no survival was found in control and *CS* flies. * indicates p<0.05.

To characterize the heritability of hyperoxia tolerance in these S_O2_A flies, we performed several experiments. First, a subset of embryos from the 28^th^ generation were collected and cultured in *normoxia* for five consecutive generations. The embryos of the 33^rd^ generation were re-introduced directly to 90% O_2_. These embryos completed their development and could be propagated perpetually in 90% O_2_, demonstrating that hyperoxia tolerance was maintained for at least five generations without further selection. Second, S_O2_A flies were crossed with *CS*, a wild-type strain sensitive to hyperoxia. As shown in Fig. 2, 66% of the pupae from this cross eclosed in 90% O_2_ as compared to 88% eclosion for S_O2_A flies and zero percent eclosion for control and *CS* flies (p<0.05). This result suggested that if the basis for hyperoxia tolerance is solely genetic, there must be more than one locus responsible for this trait (as expected) and that the mode of inheritance of at least some of the genes is a dominant one. Third, we crossed S_O2_A flies with a double balancer line (*sp/SM5; L1/TM3, Sb*) and then intercrossed the progeny (h/SM5; h/TM3 sb) to obtain h/h; h/h flies that had the 2^nd^ and 3^rd^ chromosomes (representing ∼80% of the genome) exclusively from the S_O2_A line. When embryos of h/h; h/h were exposed to 90% O_2_, 64% of pupae eclosed in hyperoxic conditions (Fig. 2) and these adult flies were able to lay eggs and propagate in 90% O_2_. The different eclosion rate between h/h; h/h flies and S_O2_A flies further suggested that the heritable hyperoxia tolerance was largely, if not exclusively, chromosomal in basis. Taken all together, these experiments indicate that genetic alterations are likely to play an important role in hyperoxia tolerance in these S_O2_A flies, although we cannot presently rule out epigenetic mechanisms.

### Hyperoxia selection changes gene expression profiles

**Figure 3:**
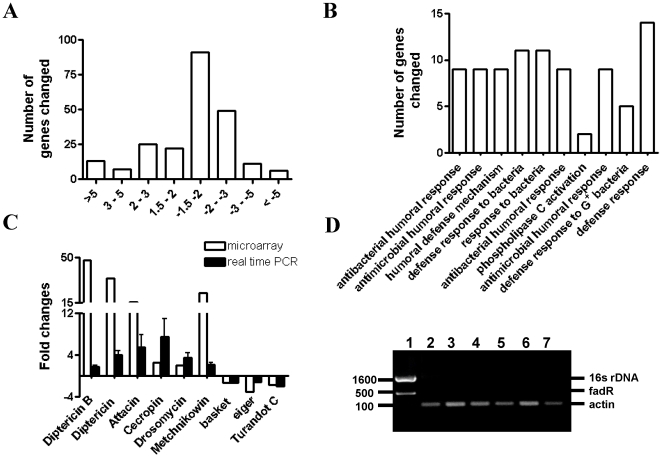
Gene expression profile changes following long-term hyperoxia selection. A) Number of genes that were significantly altered in the microarrays was summarized based on their fold changes. B) Significantly altered biological processes in the microarrays were determined by MAPPFinder and GenMAPP 2. The top 10 biological processes with the lowest p value were shown in Fig. 3B. C) Microarray data were validated by real time RT-PCR and each bar represents the average of at least three samples for each gene. D) *16s rDNA* PCR products were examined in control flies and S_O2_A flies that were cultured in the standard cornmeal agar medium. *E.coli* DNA was used as positive control; fadR and actin were used as loading control for *E. coli* and fly samples respectively. Lane 1: *E. coli* DNA; Lane 2–4: control flies; Lane 4–7: S_O2_A flies.

To identify differentially expressed genes in S_O2_A flies, we used twelve Affymetrix expression microarrays to determine the differential gene expression profiles between S_O2_A flies (cultured in 90% O_2_ chamber, n = 6) with a generation-matched control (cultured in room air chamber, n = 6). Gene expression was considered to be significantly altered if the fold change was greater than 1.5, with a Bonferroni adjusted p-value of less than 0.0001. We thus identified 227 genes that were significantly altered in expression in S_O2_A flies (Table S1); 67 of these were up-regulated and 160 were down-regulated. The distribution of fold changes in the microarrays was shown in Fig. 3A. Using MAPPFinder in conjunction with GenMAPP 2, we discovered that several gene families were significantly altered in S_O2_A flies, such as these involved in defense responses, immune response, and phospholipase C activation (Fig. 3B and Table S2). It is interesting that eight out of fourteen genes related to defense responses belong to the antimicrobial peptide (AMP) gene family, a well known gene family that plays a role in bacterial infection. Because of this finding, we first examined whether a bacterial infection induced an up-regulation of a group of AMP genes in S_O2_A flies. Using a standard approach based on PCR analysis of whole-fly homogenates with primers specific for conserved 16s rDNA sequences, we found no evidence of a microbial infection (Fig. 3D), suggesting that the up-regulation of AMP genes in S_O2_A flies was not due to the presence of bacteria. We also validated the gene expression profile of candidate genes, including AMP genes such as *Diptericin B*, *Diptericin (Dpt)*, *Attacin (Att)*, *Cecropin (Cec)*, *Drosomycin*, and *Metchnikowin* as well as stress-related genes such as *basket*, *eiger*, and *Turandot C* by real time PCR analysis and we found that real time PCR results were consistent with those of the microarrays (Fig. 3C).

### Role of single genes in hyperoxia tolerance

**Figure 4:**
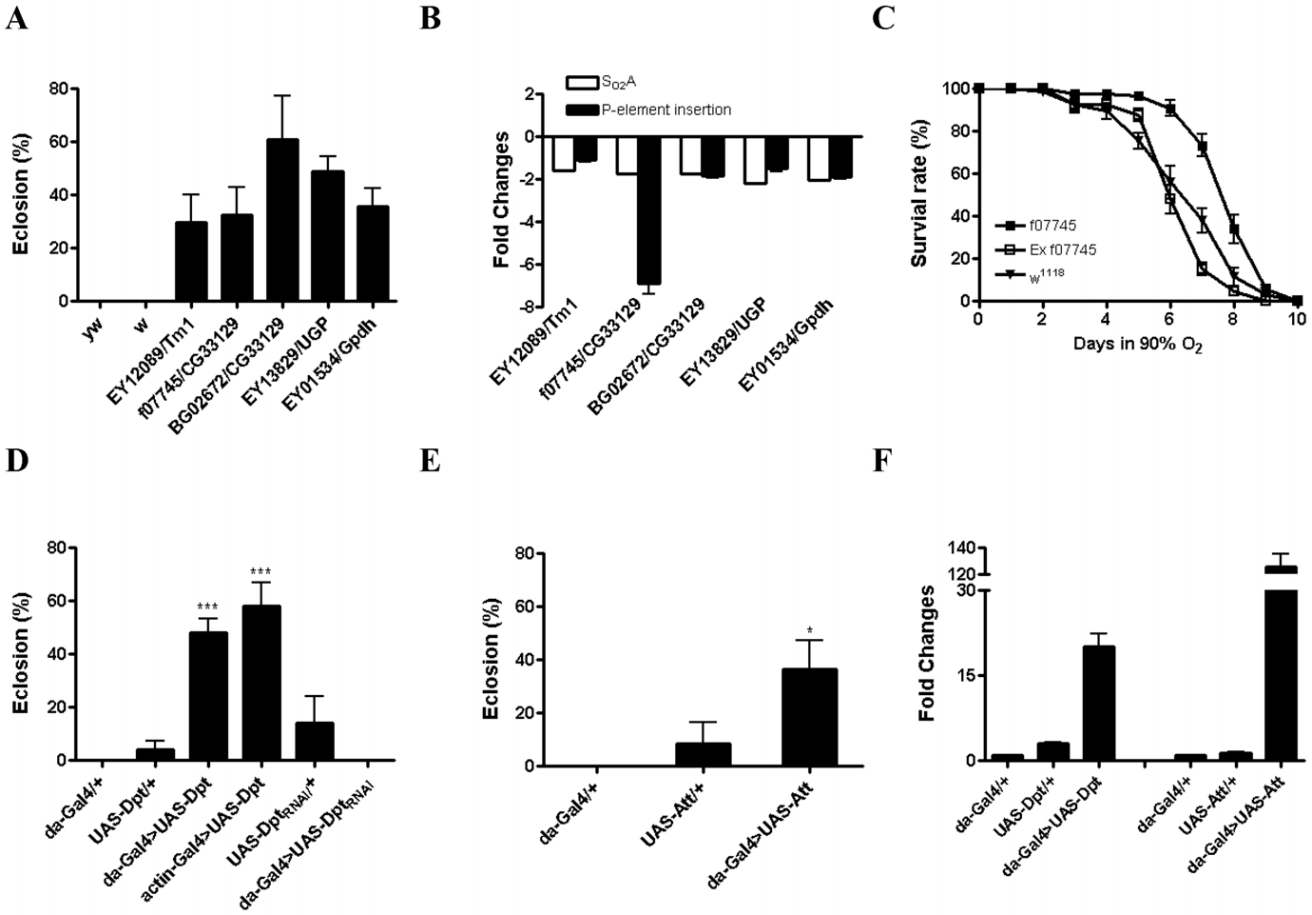
Role of single genes in hyperoxia tolerance. A) Embryos from 47 P-element insertion lines were collected and exposed to 90% O_2_ and eclosion rate was determined. Embryos from five P-element insertion lines that were able to develop into adult stage and their percent eclosion rate were shown in Fig. 4A. B) Gene expression changes in the microarrays and P-element lines, which were determined by real time PCR, were summarized in Fig. 4B. C) Adult flies with P-element insertion f07745 and without P-element insertion by precise excision (Ex f07745) were exposed to 90% O_2_ and percent survival rate was determined. Flies with precise excision were more sensitive to 90% O_2_ than flies with P-element insertion (p<0.01). D) Embryos from *da-Gal4>UAS-Dpt* and *actin-Gal4>UAS-Dpt* flies and their control lines were exposed to 90% O_2_ and eclosion rate was determined. Flies with overexpression of *Dpt* had a significantly higher eclosion rate in hyperoxia as compared to the controls (p<0.001). The percent eclosion was also determined in *da-Gal4>UAS-Dpt_RNAi_* flies and control flies as above. E) Survival of *da-Gal4>UAS-Att* flies in hyperoxia was determined as above, flies with overexpression of *Att* had a significantly higher eclosion rate than control flies (p<0.05). F) Overexpression of *Dpt* and *Att* in the *da-Gal4>UAS-AMP* flies was confirmed by real time PCR. * indicates p<0.05; ** indicates p<0.01; *** indicates p<0.001.

To investigate which individual loci with differential expression have an impact on hyperoxia tolerance and in sustaining survival in hyperoxia in the S_O2_A, a mutant screen strategy was applied. We first focused on the genes that had single P-element insertion lines in the Bloomington Stock Center, with the assumption that some such insertions would mimic the up- or down-regulation seen in the microarray data. Forty-seven P-element insertion lines (Table S3) were used in this study for 26 down-regulated genes and 13 up-regulated genes that were significantly altered in the microarrays. We tested the survival of all P-element lines in 90% O_2_ along with *yw* and *w* serving as controls. Embryos from most P-element lines and control flies stopped development and died at the larval stages. In contrast, embryos from five P-element lines EY12089, f07745 and BG02672, EY13829 and EY01534 were able to develop into the adult stage in extreme high oxygen environments and their eclosion rates were between 30% and about 60% (Fig. 4A). These P-element lines have insertions within or around genes including *Tropomyosin 1 (Tm1)*, *CG33129*, *UGP*, and *Glycerol 3 phosphate dehydrogenase (Gpdh)* respectively. The effect of P-element insertion was determined by real time PCR and these results demonstrated that these genes were down-regulated in P-element lines, which was consistent with the microarray data (Fig. 4B), suggesting that the down-regulation of these genes in S_O2_A flies played a role in sustaining survival in hyperoxia. The results of their survival were interesting since these genes were singularly down-regulated in each of the flies. To further confirm that an enhanced survival observed in these P-element lines was due to the down-regulation of these genes caused by P-element insertion, we excised one of the P-element allele f07745 from gene *CG33129*. We found that adult flies with precise excision (Ex f07745) were much more sensitive to hyperoxia and had a significant shorter life span than flies with P-element insertion (Fig. 4C, p<0.01) and embryos from this precise excision line could not develop into adult flies in 90% O_2_ (data not shown), further demonstrating the important role of this particular gene, as an example, in oxidant stress.

Given that S_O2_A flies expressed AMP genes at significantly increased levels, we also set out to explore the relationship between AMP expression and hyperoxia tolerance. We used *da-Gal4* as a driver to cross with an extensive panel of available UAS-AMP lines (*Drosocin*, *Cec*, *Att*, and *Dpt*) to generate flies with ubiquitous overexpression of each specific AMP gene. The progenies were exposed to 90% O_2_ and the eclosion rate was determined. As shown in Fig. 4D & 4E, flies with overexpression of *Dpt* and *Att* gene (*da-Gal4>UAS-AMP*) had a significantly higher eclosion rate (48% and 37% respectively) in hyperoxia as compared to the controls including *da-Gal4/+* and *UAS-AMP/+* (p<0.001 and p<0.05 respectively) lines, demonstrating that overexpression of *Dpt* and *Att* plays an important role in the survival to hyperoxia. Overexpression of *Dpt* and *Att* in the *da-Gal4>UAS-AMP* flies was confirmed by real time PCR (Fig. 4F). Using *Dpt* as an example, we found that a similar phenomenon was also observed in flies with overexpression of *Dpt* using *actin-Gal4* as a driver (Fig. 4D) and no difference in eclosion rate was found between *da-Gal4>UAS-Dpt* flies and *actin-Gal4>UAS-Dpt* flies (p>0.05). Furthermore, we decreased *Dpt* gene expression by crossing *da-Gal4* with *UAS-Dpt_RNAi_* flies and determined the survival of progeny in hyperoxia. As shown in Fig. 4D, ten percent of pupae from *UAS-Dpt_RNAi_/+* fly vials were able to eclose in 90% O_2_, in contrast, with *da-Gal4>UAS-Dpt_RNAi_* fly vials, where none was able to survive in 90% O_2_, again supporting the idea that overexpression of *Dpt* contributes to the survival in hyperoxia.

## Discussion

In the present study, we demonstrate that exposure to increased levels of O_2_ over many generations is associated with an increase in body size and weight in *Drosophila melanogaster*. This finding complements our previous study, which showed exposure to decreased O_2_ over many generations was associated with an opposite effect, i.e., a decrease in body size and weight. Thus, for example, the hypoxia-selected flies were 40% of the size of control flies at 4% O_2_ [21], while the hyperoxia-selected flies of the present study were about 20% larger than control flies at 90% O_2_. The interrelationship of O_2_ exposure and body size is interesting from many viewpoints, especially from an evolutionary perspective. Geophysical data, as well as theoretical models, have suggested that atmospheric O_2_ concentration has changed dramatically during certain metazoan periods and O_2_ changes have had a great impact on the fate and developmental characteristics of many animal species [22]. In particular, it has been postulated that Paleozoic hyperoxia helped in animal gigantism and that the subsequent hypoxic atmospheres reduced animal size [22], [23]. While these historical periods have been associated with differences in animal body size, the reported changes in O_2_ concentrations that characterized these periods may not have been large enough to affect body size to that degree. In contrast, the differences in O_2_ concentrations that led to major changes in body size and weight in our experiments were by comparison enormous.

From an experimental and clinical viewpoint, O_2_ is an important nutrient that can affect growth in invertebrates and mammals including humans, especially during periods of growth, whether at high altitude or in patients with congenital heart disease and right to left shunts [24], [25], [26]. Indeed, we and others have shown that body size in rodents and invertebrates was reduced in hypoxia [21], [22], [27], [28], [29]. It is not clear; however, why levels of O_2_ that are *higher* than normal would be associated with a larger body size, especially, in the absence of cardiopulmonary diseases. Since hemoglobin saturation does not increase with an increase in O_2_ partial pressure above normal, the increase in body size with an increased O_2_ intake may indicate that sensing mechanisms other than hemoglobin saturation play a role in growth regulation. Another possibility is that the larger body size in S_O2_A flies might have a “diluting” effect resulting in lower PO_2_ and therefore decreased hyperoxia-induced oxidative damage, leading to enhance survival in extreme high O_2_ environments.

Several microarray experiments have been done to study the effect of hyperoxia using *Drosophila* as a model [30], [31], but the tissues analyzed and the length of hyperoxia exposure differed among these studies. For example, Gruenewald and his colleagues used *Drosophila* head as samples to study gene expression profiles after 6 days of hyperoxia treatment; while Landis and his colleagues compared gene expression profiles in whole flies after 7 days of hyperoxia treatment. The comparison among these studies reveals that one gene (glutathione transferase E1, GstE1) was up-regulated in all three experiments and seventeen out of 227 genes were altered in both S_O2_A flies and the 7-day hyperoxia-treated whole fly organisms. These results, not surprisingly, suggest that hyperoxia-induced gene expression profiles are not necessarily the same when head and whole flies are studied or when acute hyperoxia is compared to long-term hyperoxia selection.

Hyperoxia selection induced changes in expression in an array of genes, but it is unlikely that all of these differentially expressed individual genes are responsible for hyperoxia tolerance in S_O2_A flies. Using a mutant screening strategy, we were able to identify some genes that functionally contributed to tolerance to extreme high oxygen environments. The idea for testing the survival of these P-element lines in high O_2_ (i.e., 90% O_2_) directly was that such P-element mutations, if present in genes of interest (based on the microarray results), could be either tolerant or sensitive to hyperoxia treatment, depending on whether that particular gene is important in the phenotype. Almost 90% of P-element lines (42 out of 47 lines) that we have currently tested can not survive and do not finish their life cycle in 90% O_2_. There are potentially several reasons for the lack of survival of the overwhelming majority of the P-element lines. We speculate that a) the changes in expression of a particular gene may not be sufficient for an effect; and b) the change in expression may not contribute at all to hyperoxia tolerance. For instance, some of these gene changes in the microarrays may be due to a consequence of ‘hitchhiking’ through genetic linkage. However, we show that five P-element lines including EY12089, f07745 and BG02672, EY13829, and EY01534, tolerate hyperoxia directly, i.e., without being exposed to the gradually increasing O_2_ concentrations from 60 to 90% O_2_ over many generations, as in the selected flies. These P-elements were inserted in *Tm1*, *CG33129*, *UGP and Gpdh* genes respectively. Tm1 is an actin-binding protein that stabilizes microfilaments and has function in regulating muscle contraction [32], [33]. Tm1 expression is increased in the choroid plexus of Alzheimer Disease patients [34] and in glaucomatous cells that contain higher levels of ROS [35]. Furthermore, Kubo et al have shown that increased Tm1 alpha mRNA and protein level in peroxiredoxin 6 (Prdx6)-deficient lens epithelial cells (LECs) was associated with increased vulnerability to UV-B radiation, increased ROS expression and apoptosis, as compared with wild type LECs [36]. These observations suggest that decreased *Tm1* mRNA in the P-element insertion line is associated with a lower ROS expression and a better survival in hyperoxia. *Drosophila* Gpdh has two well-characterized metabolic functions, including bridging glycolysis and triglyceride metabolism and serving in the glycerol-3-phosphate shuttle to regenerate NAD+ from NADH in mitochondria [37]. Xun and his colleagues have suggested that an increased level of Gpdh in α-synuclein expressing (Parkinson's disease like) flies raises the possibility that cells undergo increased oxidative phosphorylation, which would generate more ROS that damages cells [38]. The ortholog of *CG33129* in humans, named transmembrane protein 214, is involved in protein binding. *UGP* has UDP glucose pyrophosphorylase activity, which is a key enzyme producing UDP-glucose, and is involved in metabolic pathways such as sucrose and cellulose synthesis. UGP is believed to be involved in the homeostatic readjustment of plant responses to environmental signals, such as cold and light [39]. It is not clear whether decreased *CG33129* and *UGP* gene expression contributed to survival in hyperoxia-induced oxidative stress directly or indirectly, but these identified genes could provide the first step towards a better understanding of the mechanisms of hyperoxia tolerance. Because of conservation of genes between *Drosophila melanogaster* and mammals [17], we believe that such genes that contribute to hyperoxia tolerance in *Drosophila* may also be critical in hyperoxia adaptation in physiological or pathological conditions in mammals.

One of the most strongly up-regulated gene families in our hyperoxia-tolerant strain was the AMP gene family. Using UAS/Gal4 system, we overexpressed several AMP genes and determined the survival of the progeny in hyperoxia. We found that increased *Dpt* and *Att* gene expression enhanced survival in 90% O_2_, demonstrating that *Dpt* and *Att* play a role in hyperoxia tolerance. Overexpression of *Dpt* and *Att* alone have been shown to contribute to resistance to some Gram-negative bacteria and fungi such as *E.coil* and *F. oxysporum* [40]. Our study, for the first time, demonstrates that overexpression of these two AMP genes has a protective role in the survival to hyperoxia. It is well known that the oxidant burst in the white blood cells is important in killing invading bacteria [41]. An intriguing idea would be that overexpression of AMP genes not only protects the host against bacterial infections but also protects the host against white blood cells. Although it is not clear whether AMP gene overexpression could attenuate O_2_ burst-induced tissue injury in sepsis and fulminant bacterial invasion, recent data have suggested that, besides their antimicrobial function, some AMP genes have multiple roles in inflammation, proliferation, wound healing, chemotaxis, and antiapoptosis [42], [43], [44], [45], [46]. For example, PR-39, a cathelicidin with antimicrobial properties, attenuates the apoptotic response of apoptosis-inducing drugs such as etoposide, bleomycin, tert-butylhydroperoxide and 2-deoxy-d-ribose [47]. Chen et al. and Suttmann have demonstrated that cecropin are highly potent against bacteria and cancer cells but not normal cells [48]. Based on their observations, Papo and Shai proposed that AMPs, which are positively charged molecules, may “recognize” negatively charged targets such as cancer cells and bacteria [49]. We also raise the possibility here as to whether cationic AMP peptides such as Diptericin and Attacin may trap or “scavenge” free radical anions and attenuate oxygen toxicity because of their surface charge [50], [51].

In conclusion, we have succeeded in generating a *Drosophila* strain that can live perpetually at extremely high O_2_ (90% O_2_), a level lethal to naïve flies. This tolerance is an inherited trait as result of a long-term experimental selection. The down-regulation of *Tm1*, *CG3312*, *UGF*, and *Gpdh*, and the up-regulation of *Dpt* and *Att* play a crucial role in hypoxia tolerance and survival in extreme high O_2_ conditions.

## Materials and Methods

### Parental *Drosophila melanogaster* isogenic lines

The long term experimental selection experiment was performed as described previously [21] with minor modifications. Isogenic *Drosophial melanogaster* lines (n = 27; kindly provided by Dr. Andrew Davis) were intercrossed to provide allelic variation for laboratory selection. Male and virgin female flies (n = 20) from each of the 27 lines were collected and pooled in each chamber and maintained at room temperature. Embryos from this pooled fly population were collected as F1 and subjected to hyperoxia or normoxia respectively. Percent eclosion (adult emergence) was determined by calculating the ratio of the number of empty pupae to the number of total pupae in each culture vial.

### Culture chambers

Population chambers (26cm×16cm×16cm) were specially designed for the laboratory selection experiments. These chambers were supplied with either O_2_ balanced with N_2_ for each O_2_ concentration (hyperoxia) or to normoxic room air (21% O_2_, for control flies). Humidity was maintained by passing the gas through water prior to entry into the chambers. The flow speed was monitored with a 565 Glass Tube Flowmeter (Concoa, Virginia Beach, VA), and the O_2_ level within the chamber was periodically tested with a Diamond General 733 Clark Style electrode (Diamond General Development Corp., Ann Arbor, MI).

### Phenotypic assays

Several assays were used to determine the phenotypic differences between S_O2_A and control flies. **1. Body weight measurement**. Three sets of 100 male flies were collected from each chamber and weighed. **2. Wing area and cell number measurement**. Wings from male flies of each group (n = 12) were collected, flattened on glass slides and mounted with Permount. The images of the wings were digitized using an Axiovert 200 M microscope (Carl Zeiss MicroImaging, Inc., Thornwood, NY, USA) with an image capturing Axiovision software program at a magnification of 10× and 40×. The microchaetae from the whole field were overlaid using Adobe Photoshop 9.1 and counted using Image J (Version 1.42n). **3. Survival in hyperoxia**. Embryos were cultured in normoxia for 48 hrs and then transferred to 90% O_2_. The percent eclosion was determined after 3 weeks. Three-to-five day old adult flies were exposed to 90% O_2_ using a similar chamber as in the selection experiment. Percent survival was scored daily. Experiments were repeated at least 4 times for each line.

### RNA extraction and microarray analysis

Twelve samples from hyperoxia-selected adult flies (S_O2_A in 90% O_2_ chamber, n = 6) and control flies (in room air chamber, n = 6) were used for microarray experiments, in which had a pool of 25 male and 25 female flies from each chamber. Total RNA was extracted as previously described [21]. Affymetrix *Drosophila* Genome 2.0 arrays were used and probe labeling, array hybridization, and image scanning were performed following the standard protocol according to manufacturer's instruction (Affymetrix, Santa Clara, CA). The resulting data files from the Affymetrix scanner were background-subtracted and normalized using “Model-Based Expression Indexes” (MBEI) available in dChip and the “Robust Multi-array Average” (RMA) software [52]. The alterations in global gene expression in the context of specific biologic pathways and molecular functions were determined using GenMAPP 2.0 software [53]. The microarray data were MIAME compliant and data of microarray analyses can be retrieved under the series access number GSE12160 in the National Institute for Biotechnology Information (http://www.ncbi.nlm.nih.gov/geo).

### Fly stocks and culture

*Canton-S* (*CS*), *yw*, *w*, *da-Gal4* and all P-element lines tested here were obtained from the Bloomington Stock Center. *sp/SM5; L1/TM3, Sb* flies were generously provided by Dr. Steven Wasserman (University of California San Diego, USA). *UAS-AMP* flies (*w*; *imd*, *UAS-AMP*; *spz^rm7^*, *UAS-AMP*/*TM6C*) were generously provided by Dr. Bruno Lemaitre (Global Health Institute, Switzerland). *UAS-Diptericin_RNAi_* (*UAS-Dpt_RNAi_*) flies were obtained from the Vienna Drosophila RNAi Center. All stocks were maintained on standard cornmeal agar medium.

### Real time RT-PCR

Total RNA was treated with DNase I (Ambion, Austin, TX). cDNA synthesis from 1 µg of total RNA was performed using SuperScript First-Strand Synthesis System for RT-PCR (Invitrogen, Carlsbad, CA). Real time RT-PCR was performed using Power SYBR Green PCR Master Mix (Applied Biosystems, Foster city, CA) following a standard protocol recommended by manufacturers. Primers used in the present study were shown in Table S4. Results were analyzed by ABI Prism 7900 Sequence Detection System (Applied Biosystems, Foster city, CA). Relative gene expression was calculated after normalization to β-Actin-5C.

### P-element excision

To excise the P-element in the *w^1118^; PBacCG33129^f07745^* line, male P-element insertion flies were crossed to female virgins that express the Δ2–3 transposase. Male or female progeny possessing both the *P*-element and the transposase were then individually crossed to females or males with the 2^nd^ chromosome balancer *cyo*. Following screening for loss of eye color, several independent excision lines were established. Precise excision was confirmed by sequencing a PCR product of the genomic region spanning the P-element insertion site with primers (fwd: aatggtttctcctgcattgg; and rev:agaacggcggaatacacaac).

### Statistics

Kaplan-Meier survival analysis was used to compare life span between groups; all other data were analyzed using student t-test or one-way ANOVA and graphed using GraphPad Prism 4.02 (GraphPad Software, Inc., San Diego, CA). Results were expressed as group mean ± SEM. Difference in means were considered statistically significant when p<0.05, unless otherwise stated.

## Supporting Information

Table S1 Summary of Significant Alteration in Gene Expression in Hyperoxia-Selected Drosophila melanogaster.
(0.05 MB XLS)

Table S2 Summary of Significant Changes in GO-based Gene Expression Profiles in Hyperoxia-Selected Drosophila melanogaster.
(0.03 MB XLS)

Table S3 Survival of P-element Insertion lines and da-Gal4>UAS-AMP lines in 90% O2 (Score: 0, no survival; 1, reaching larvae stage; 2, reaching pupae stage; 3, reaching adult stage).
(0.03 MB XLS)

Table S4 List of Primers Used in the Current Studies.
(0.02 MB XLS)
